# Whole-genome survey and phylogenetic analysis of *Gadus macrocephalus*

**DOI:** 10.1042/BSR20221037

**Published:** 2022-07-15

**Authors:** Yiqing Ma, Fangrui Lou, Xiaofei Yin, Bailin Cong, Shenghao Liu, Linlin Zhao, Li Zheng

**Affiliations:** 1 School of Advanced Manufacturing, Fuzhou University, Jinjiang 362200, China; 2 First Institute of Oceanography, Ministry of Natural Resources, Qingdao 266100, P.R. China; 3 School of Ocean, Yantai University, Yantai 264005, China

**Keywords:** demographic history, Gadus macrocephalus, genome survey, phylogenetic analysis

## Abstract

*Gadus macrocephalus* (Pacific cod) is an economically important species on the northern coast of the Pacific. Although numerous studies on *G. macrocephalus* exist, there are few reports on its genomic data. Here, we used whole-genome sequencing data to elucidate the genomic characteristics and phylogenetic relationship of *G. macrocephalus.* From the 19-mer frequency distribution, the genome size was estimated to be 658.22 Mb. The heterozygosity, repetitive sequence content and GC content were approximately 0.62%, 27.50% and 44.73%, respectively. The draft genome sequences were initially assembled, yielding a total of 500,760 scaffolds (N50 = 3565 bp). A total of 789,860 microsatellite motifs were identified from the genomic data, and dinucleotide repeat was the most dominant simple sequence repeat motif. As a byproduct of whole-genome sequencing, the mitochondrial genome was assembled to investigate the evolutionary relationships between *G. macrocephalus* and its relatives. On the basis of 13 protein-coding gene sequences of the mitochondrial genome of Gadidae species, the maximum likelihood phylogenetic tree showed that complicated relationships and divergence times among Gadidae species. Demographic history analysis revealed changes in the *G. macrocephalus* population during the Pleistocene by using the pairwise sequentially Markovian coalescent model. These findings supplement the genomic data of *G. macrocephalus*, and make a valuable contribution to the whole-genome studies on *G. macrocephalus*.

## Introduction

**Figure 1:**
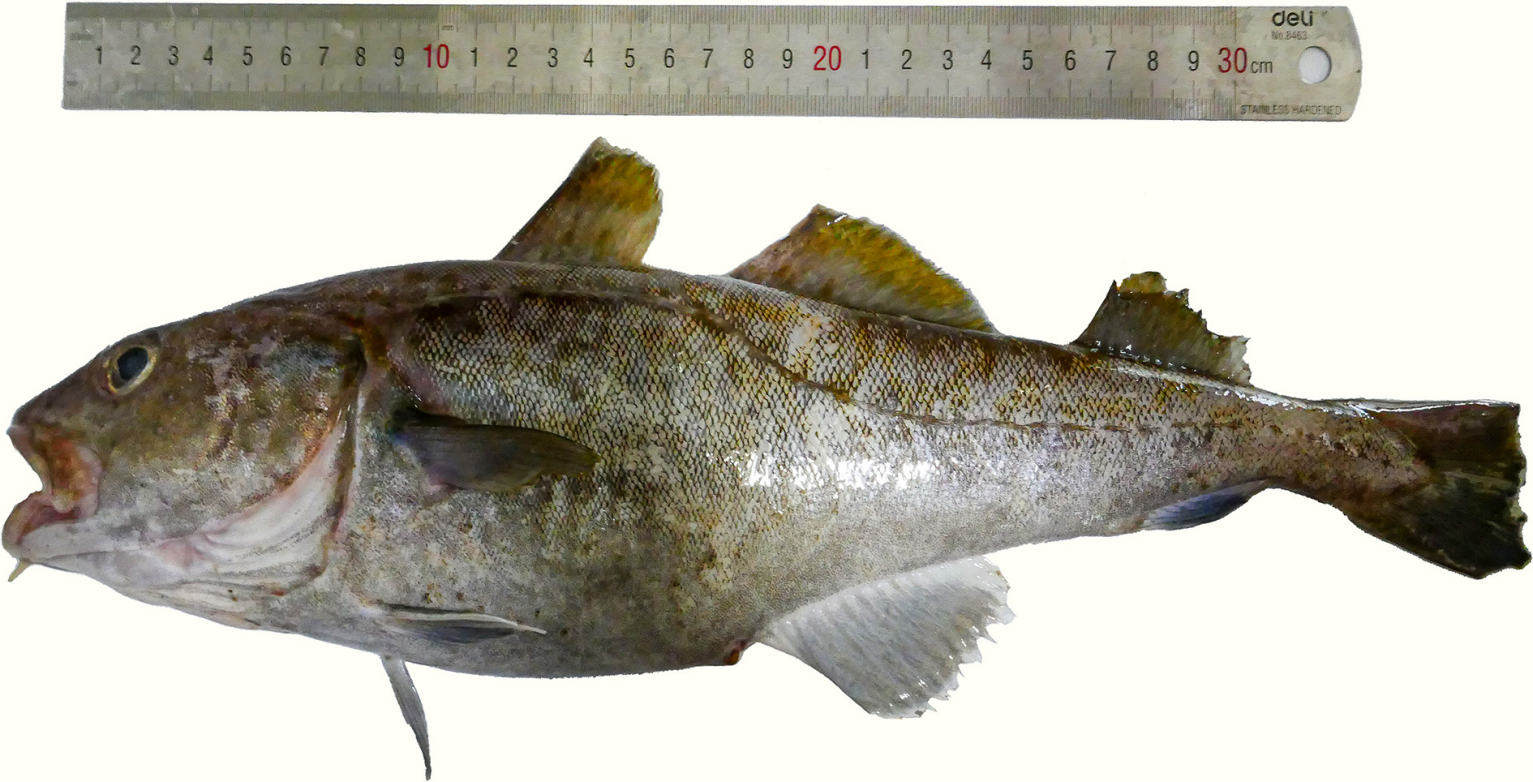
The morphological picture of *G. macrocephalus* *G. macrocephalus* belongs to the order Gadiformes, Gadidae and *Gadus*.

*Gadus macrocephalus* (Pacific cod) belongs to the order Gadiformes, Gadidae (Figure 1). It is mainly distributed in the northern coast of the Pacific, from the Yellow Sea of China through the Sea of Japan and Okhotsk and Bering Seas to California in the eastern North Pacific [1,2]. The species is economically important throughout its distribution range. However, the age structure of *G. macrocephalus* from the Yellow Sea of China is relatively simple, and its resources are easily affected by changes in the external environment [3]. In recent years, owing to human overfishing and environmental variations, the total number of catches of *G. macrocephalus* in Chinese waters has declined [4]. At present, research on *G. macrocephalus* mainly focuses on its chemical composition [5], migratory patterns [6], population genetics [7], feeding [8] and immune responses [9]. Complete mitochondrial genome sequences are available for this fish [10], but the genome sequences relatively less are known, which will be immensely useful for the management of genetic resources.

With the rapid development of sequencing technology, whole-genome sequencing (WGS) has been widely applied in genomic studies of fish species [11,12]. Genome survey analysis using WGS data was performed in a number of species to obtain fundamental genomic information, viz. genome size, heterozygosity levels, repetitive sequence content and GC content [13,14]. Information on genome-wide microsatellite markers as well as mitochondrial genomes, obtained from genomic data, could be helpful for population genetics and evolutionary analysis. In addition, WGS data can also provide better insight into the demographic history of a population [15].

In this report, WGS data were employed to investigate the genomic characteristics of *G. macrocephalus*. The mitochondrial genome of *G. macrocephalus* was assembled. Moreover, phylogenetic relationships, microsatellite motifs and demographic history inference were also carried out. The genomic data and phylogenetic analysis will be significant for genetic research in the future.

## Materials and methods

### DNA extraction and sequencing

**Figure 2:**
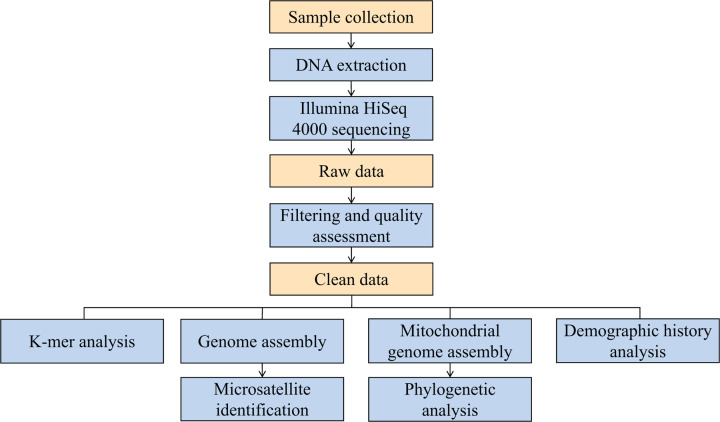
Overview of the experimental design and analysis pipeline Bioinformatics workflow for *K*-mer analysis, genome assembly, microsatellite identification, mitochondrial genome assembly, phylogenetic and demographic history analysis.

The experimental design, sequencing and data analysis pipeline were showed (Figure 2). We collected muscle tissues from one *G. macrocephalus* in the fishing port, Qingdao, Shandong Province, China, in December 2020. Then, the muscle tissue was quickly frozen in liquid nitrogen for 1 h before storage at −80°C. The DNeasy Blood and Tissue Kit (Qiagen, Germany) was used to extract total genomic DNA from the muscle tissue following the manufacturer’s instructions. For sequencing, DNA libraries were constructed with insert sizes of 350 bp, and paired-end sequencing was performed on the Illumina HiSeq 4000 platform. The sequence data were deposited in the Short Read Archive (SRA) database (http://www.ncbi.nlm.nih.gov/sra) under accession number PRJNA793360.

### Genome assembly and identification of microsatellite motifs

To produce clean data, the raw data from WGS were filtered by Fastp 0.20.1 [16], removing the adaptors, ambiguous reads and low-quality reads. To assess the characteristics of the *G. macrocephalus* genome, we used the clean data for *K*-mer analysis (*K* = 19) in Jellyfish software [17]. From the results of *K*-mer analysis, we obtained the size, heterozygosity and repetitive sequence content of the genome. Genome size was estimated by using the equation Genome size = K-mer_num / peak_depth, where K-mer_num is the total number of best predicted *K*-mers and peak_depth is the expected value of the *K*-mer depth [17]. After that, genome size was revised by excluding *K*-mer errors via the formula Revised genome size = Genome size × (1 − Error rate) [17].

To obtain the genomic sequence, we used SOAPdenovo software [18] to assemble the genome. Before assembly, we excluded clean reads with a length <100 bp to generate a high-quality genome. Clean reads were assembled into contigs by using the de Bruijn graph algorithm [18]. On the basis of their pair-end relations, contigs were connected into scaffolds, with ‘*N*’ representing unknown sequences between two contiguous contigs [18].

To search for simple sequence repeats (SSRs) in the *G. macrocephalus* genome, we used the Perl script MIcroSAtellite (MISA, http://pgrc.ipk-gatersleben.de/misa) to identify microsatellite motifs. The parameters were set as the detection of mono-, di-, tri-, tetra-, penta- and hexanucleotide SSR motifs with a minimum of 10, 6, 5, 5, 5 and 5 repeats, respectively [19].

### Mitochondrial genome assembly, phylogenetic and demographic history analysis

To obtain the 13 protein-coding gene sequences of mitochondrial genome, the clean reads from Fastp software were used to construct the mitochondrial genome via MitoFinder v.1.4 [20]. MitoFinder employs the MEGAHIT method [21] for mitochondrial genome assembly. We used the mitochondrial genome sequence of *G. macrocephalus* in the NCBI GenBank database (accession number: NC036931) as the reference. Then, these contigs were used as a guide for structural annotation of the mitochondrial genome in MitoFish [22].

To investigate the phylogenetic relationships and divergence times of Gadidae species (especially for intraspecific relationships), we downloaded 13 protein-coding gene sequences of the mitochondrial genome of Gadidae species and an outgroup (*Bregmaceros nectabanus*, smallscale codlet) from the GenBank database. A phylogenetic tree was drawn by the maximum likelihood algorithm with 1000 replicates in MEGA 11 [23]. Additionally, the divergence time analysis of Gadidae species was performed with the clocks tool of MEGA 11. We selected three calibration points from the TimeTree tool (http://www.timetree.org) [24] to determine the divergence times: (1) *G. macrocephalus* and *Gadiculus thori* (silvery pout) diverged 25.90 million years ago (Mya), (2) *G. macrocephalus* and *Melanogrammus aeglefinus* (haddock) diverged 10.90 Mya, and (3) *G. macrocephalus* and *Boreogadus saida* (polar cod) diverged 8.40 Mya.

To infer the demographic history of *G. macrocephalus*, the whole genome of *G. morhua* (Atlantic cod; accession number: GCA_902167405.1) was retrieved from the NCBI database and used as a reference genome. Then, the clean reads of *G. macrocephalus* were aligned to the reference genome of *G. morhua* by using the bwa-mem algorithm in Burrows–Wheeler Aligner (BWA) [25]. We estimated the history of effective population sizes (*N_e_*) using the pairwise sequentially Markovian coalescent (PSMC) model [15]. The PSMC analysis command included the maximum of 25 model iterations (-N25), the upper limit for the most recent common ancestor (-t15), ‘-r5’ for the initial θ/ρ, and ‘-p 4+25*2+4+6’ atomic intervals. The reconstructed demographic history was plotted using the ‘psmc_plot.pl’ script with a generation time of *g* = 6 years and a mutation rate of μ = 8.29 × 10^−9^ site^−1^ year^−1^ (5.0 × 10^−8^ site^−1^ generation^−1^) [26]. By default, the variance of *N_e_* was estimated using 100 bootstrap replicates.

## Results

### Genome assembly and identification of microsatellite motifs

A total of 56.63 Gb raw data were generated by the Illumina HiSeq platform (Table 1). After filtering and quality assessment, the clean data amounted to approximately 53.94 Gb. The Q20 (97.19%) and Q30 (93.08%) were over 90%, and the GC content was approximately 44.73%. These data indicated that the clean data were sufficient to capture most of the genomic information.

**Table 1:** Quality control information of sequencing data

Raw data (Gb)	Clean data (Gb)	Q20(%)	Q30(%)	GC content (%)
56.63	53.94	97.19	93.08	44.73

**Figure 3:**
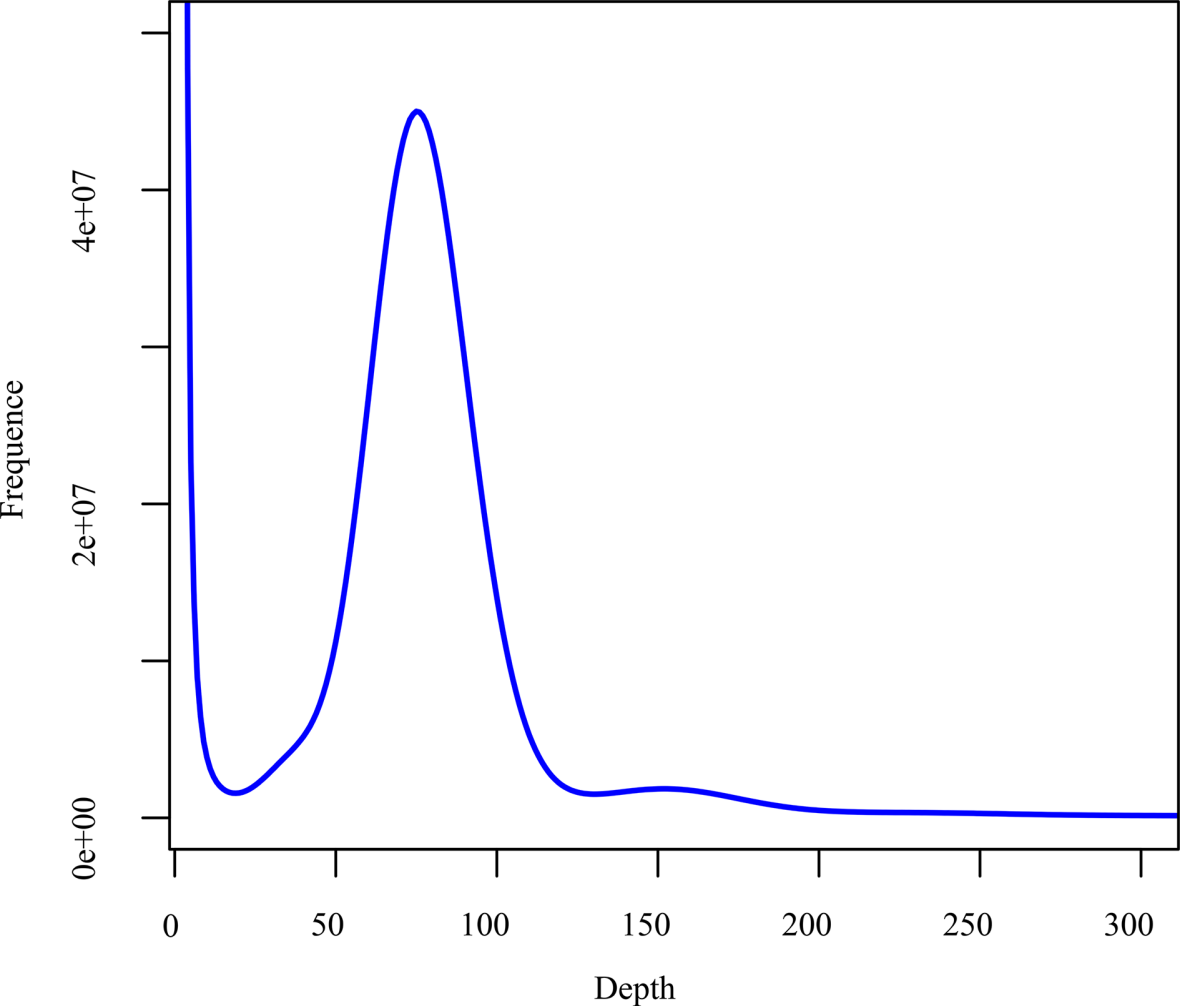
*K*-mer (*K* = 19) analysis for estimating the genome size of *G. macrocephalus* The *x*-axis represents depth and the *y*-axis indicates the proportion of the frequency at that depth.

Jellyfish software was used for *K*-mer analysis. As a result, the peak of the 19-mer frequency distribution was 69, and the total number of *K*-mers was 47,386,140,246 (Figure 3). After removing *K*-mer errors, the results of *K*-mer analysis showed that the revised genome size of *G. macrocephalus* was approximately 658.22 Mb. In the *K*-mer distribution, there was no obvious heterozygous peak, and the heterozygosity was approximately 0.62%. The repetitive sequence content of *G. macrocephalus* was 27.50%.

The clean reads were used to carry out *de novo* assembly based on *K*-mer analysis. Table 2 showed the overall statistics of genome assembly. After assembly, a total length of 532,671,928 bp contigs were obtained with the contig N50 value of 867 bp and N90 value of 154 bp, and the maximum contig was 36,036 bp in length. A total of 1,187,811 contigs were obtained, and only the length of 119 contigs >10 kb. The number of scaffolds was 500,760 and the total length of scaffolds was approximately 527,441,282 bp, and gaps accounted for 3.02% of the total length of scaffolds. The scaffold N50 value of 3565 bp and N90 value of 424 bp, and the maximum scaffold was 66,220 bp in length.

**Table 2:** Statistics of the *G. macrocephalus* assembled genome

	Total length (bp)	Total number	N50 length (bp)	N90 length (bp)
**Contigs**	532,671,928	1,187,811	867	154
**Scaffolds**	527,441,282	500,760	3565	424

**Figure 4:**
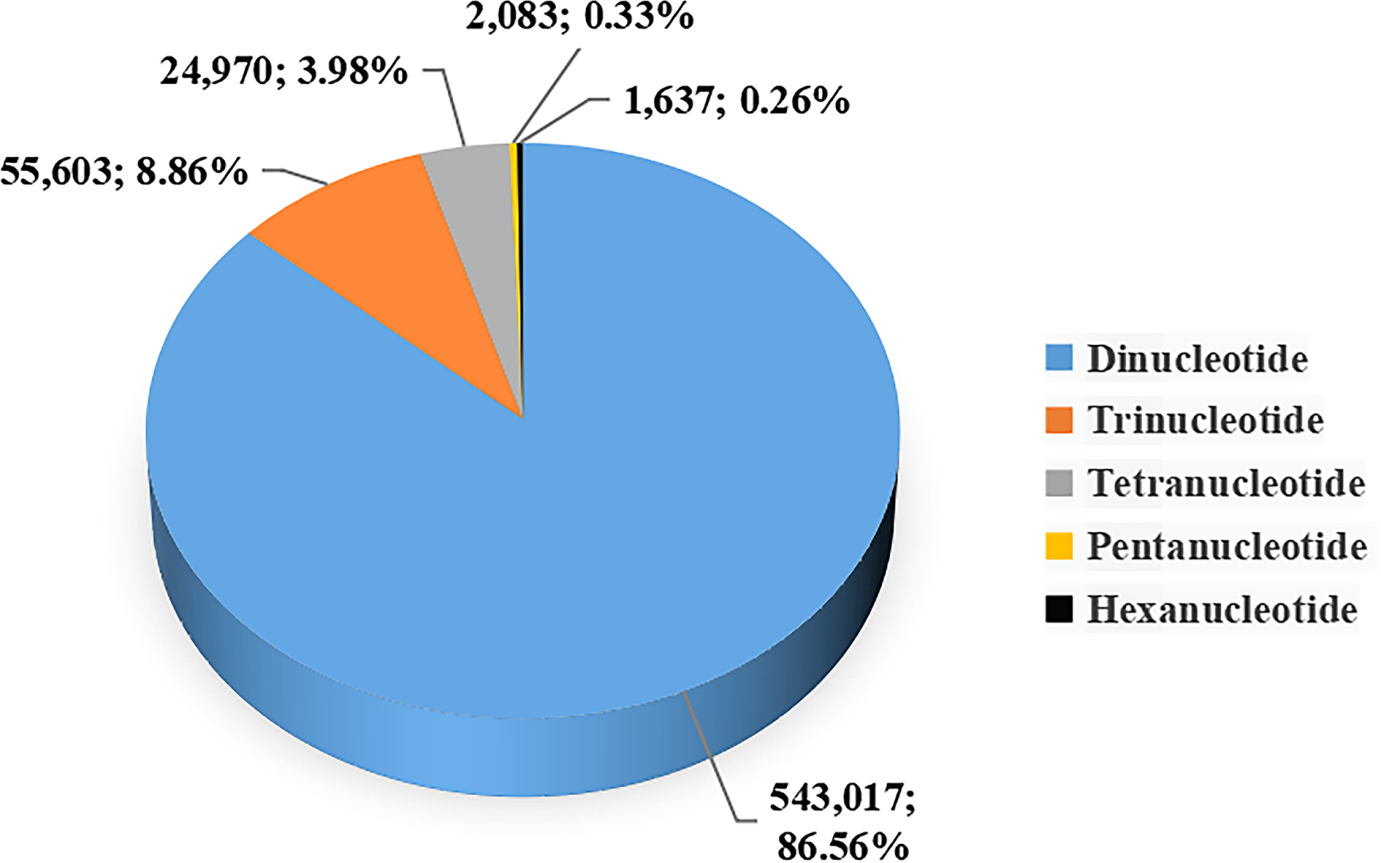
Frequency of identified microsatellite motif types The proportions of di-, tri-, tetra-, penta- and hexanucleotide microsatellite motifs in the SSRs of *G. macrocephalus*.

**Figure 5:**
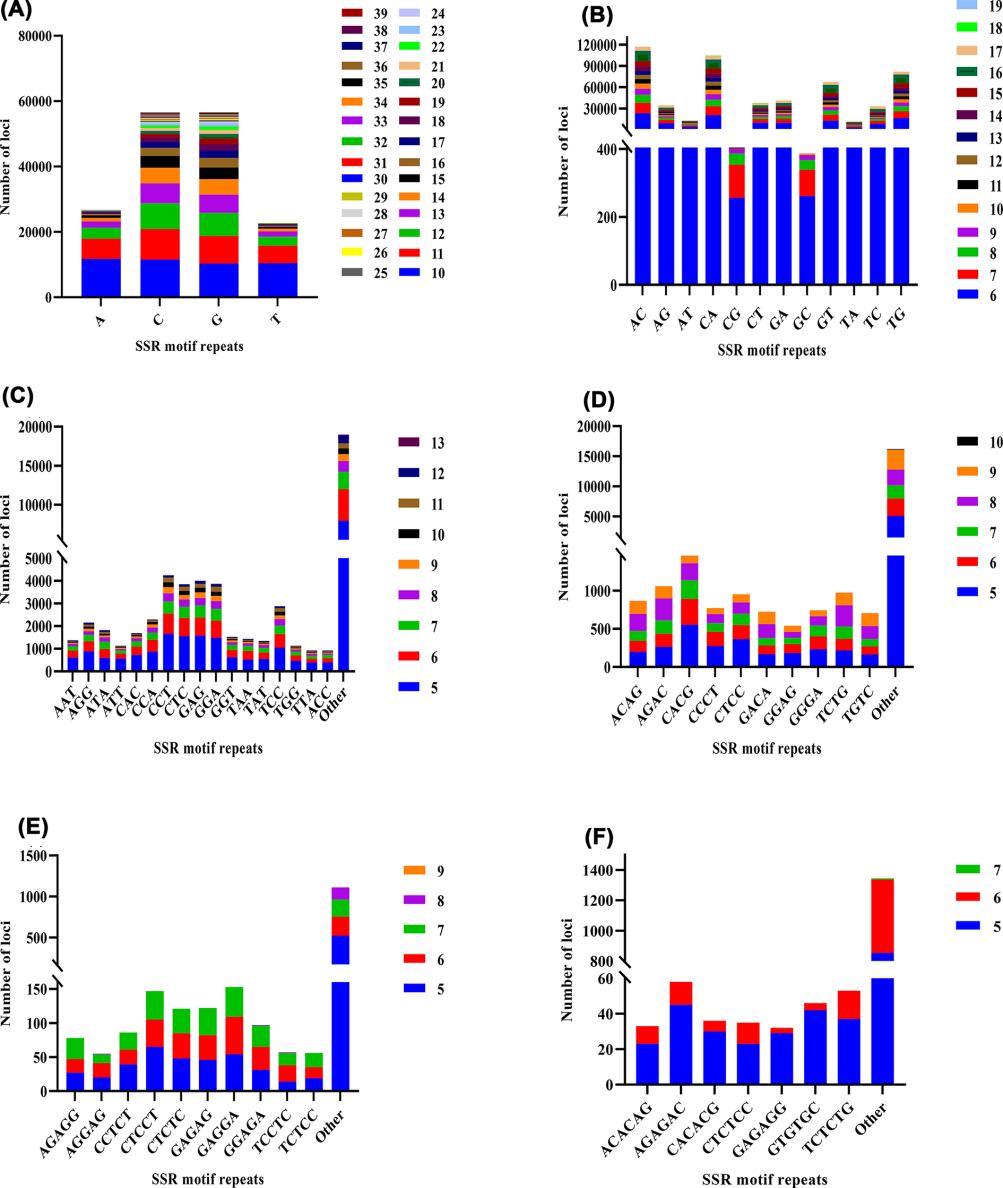
The distribution and frequency of microsatellite motifs (**A**) Frequency of different mononucleotide microsatellite motifs. (**B**) Frequency of different dinucleotide microsatellite motifs. (**C**) Frequency of different trinucleotide microsatellite motifs. (**D**) Frequency of different tetranucleotide microsatellite motifs. (**E**) Frequency of different pentanucleotide microsatellite motifs. (**F**) Frequency of different hexanucleotide microsatellite motifs.

SSRs were identified from the draft genome sequences by MISA. A total of 500,760 sequences with a total length of 527,441,282 bp were examined, and 789,860 SSRs were finally identified (Table 3). In the dinucleotide to hexanucleotide microsatellite motifs, the motif types of microsatellites were 86.56% (543,017) dinucleotide, 8.86% (55,603) trinucleotide, 3.98% (24,970) tetranucleotide, 0.33% (2083) pentanucleotide and 0.26% (1637) hexanucleotide repeats (Figure 4). The distribution and frequency of different motifs in mono-, di-, tri-, tetra-, penta- and hexanucleotide repeats were also showed (Figure 5). Among the dinucleotide microsatellite motifs, the AC (117,131) repeat motif was the most abundant, followed by CA (104,460) (Figure 5B). Within the trinucleotide repeat motifs, the major repeat motifs were CCT (4244) and GAG (4008) (Figure 5C). Additionally, among the tetranucleotide repeat motifs, the common motifs were CACG (1459) and AGAC (1059) (Figure 5D). The GAGGA (153) (Figure 5E) repeat motif was the most frequent pentanucleotide repeat, and the AGAGAC (58) (Figure 5F) was the most frequent hexanucleotide repeat.

**Table 3:** Statistics of the microsatellite recognition results

Statistical items	Numbers
Total number of sequences examined	500,760
Total size of examined sequences (bp)	527,441,282
Total number of identified SSRs	789,860
Number of SSR-containing sequences	236,221
Number of sequences containing more than 1 SSR	150,400
Number of SSRs present in compound formation	194,882

### Mitochondrial genome assembly, phylogenetic and demographic history analysis

**Figure 6:**
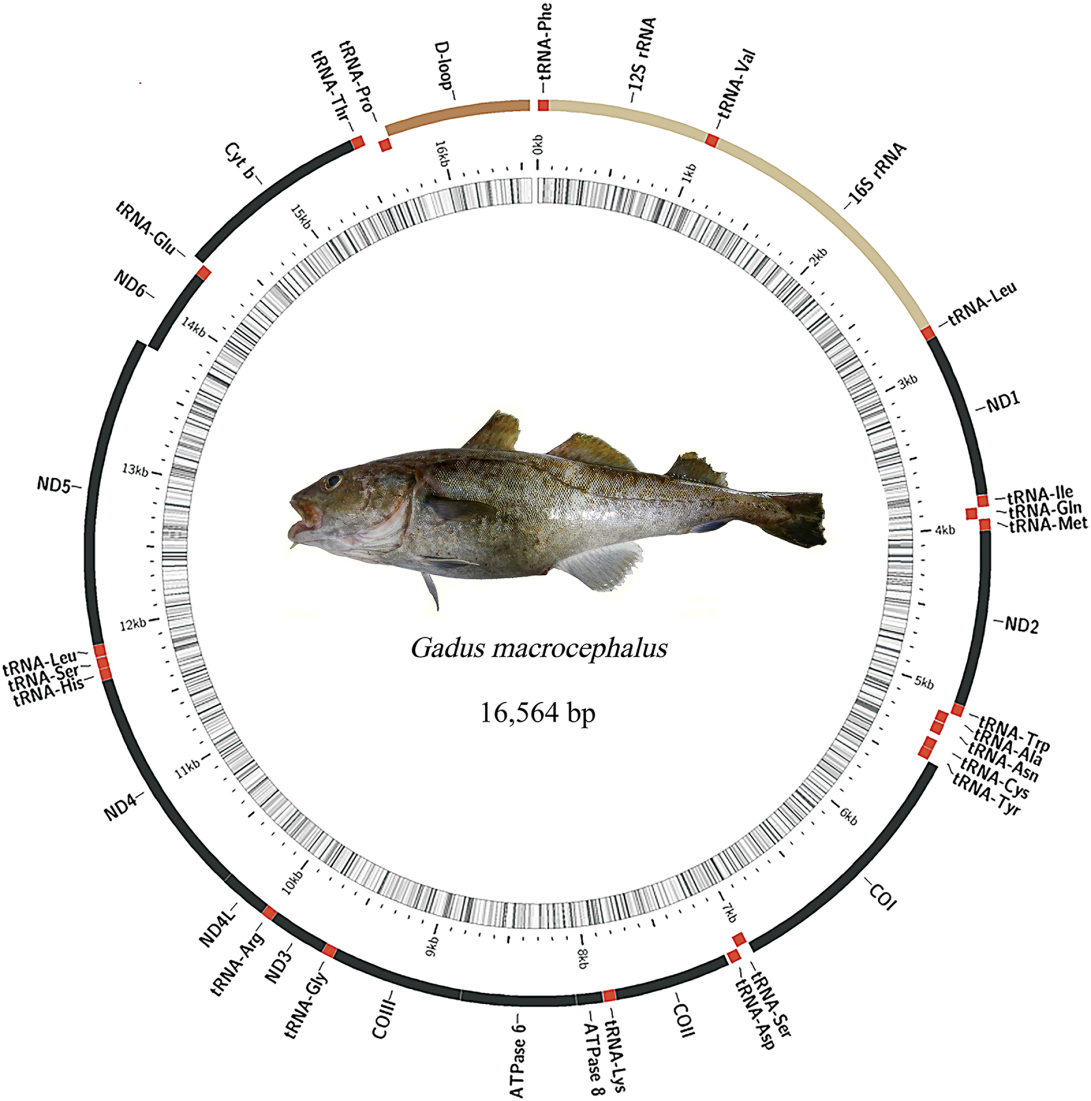
Mitochondrial genome of *G. macrocephalus* In the inner circle, the dark and light shaded areas indicate the GC and AT contents of the mitochondrial genome, respectively. In the outer circle, 37 genes on the H-strand and L-strand are shown outside and inside, respectively. tRNA genes (red areas) are named using three-letter amino acid abbreviations.

Mitochondrial genome assembly yielded a closed circular DNA molecule with a length of 16,564 bp (accession number: OM174271). A comparison of the lengths of the mitochondrial genomes of *G. macrocephalus* from previously reported and assembled in this study, revealed that they were extraordinarily similar (a difference of only 3 bp), and the identity of these two mitochondrial genome sequences reached 100%. These two mitochondrial genomes contained the same set of 37 genes, including 13 protein-coding genes, 22 tRNA genes and 2 rRNA genes. Among them, 8 tRNA genes (tRNA-Pro, tRNA-Gln, tRNA-Ala, tRNA-Asn, tRNA-Cys, tRNA-Tyr, tRNA-Ser and tRNA-Glu) and ND6 were located in the light chain, and the others were located in the heavy chain. Besides, structural annotation of the mitochondrial genome indicated that it was composed of a coding region and a D-loop (Figure 6). The D-loop was located between tRNA-Pro and tRNA-Phe with location ranging from 1 to 53 bp and 15,752 to 16,564 bp.

**Figure 7:**
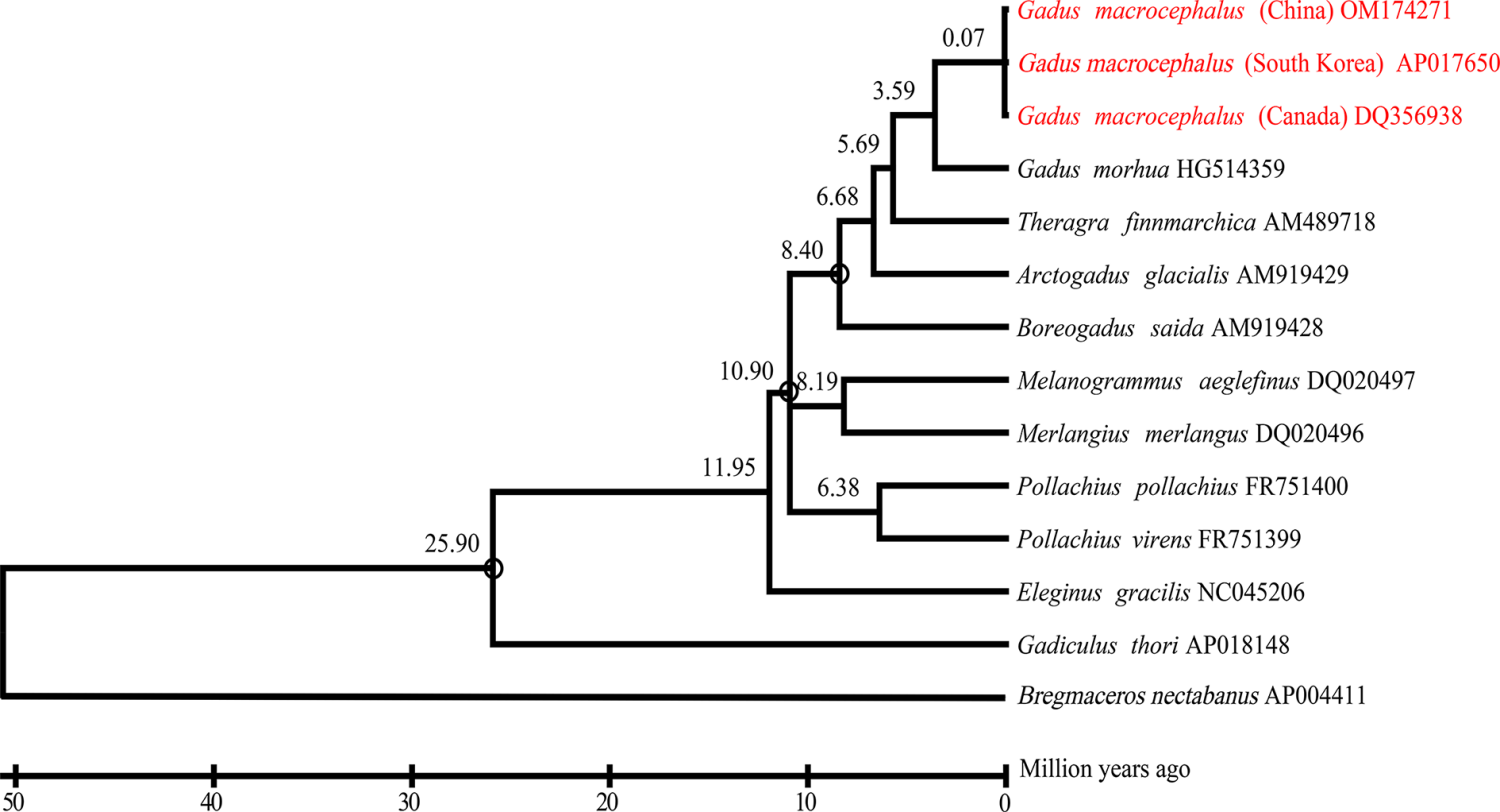
Phylogenetic analysis of *G. macrocephalus* A maximum likelihood phylogenetic tree of Gadidae species obtained from MEGA 11 based on the 13 protein-coding gene sequences of the mitochondrial genome. The mean divergence time of the nodes is shown next to the nodes. Three black circles represent three calibration points.

A maximum likelihood phylogenetic tree was constructed based on the 13 protein-coding gene sequences of the mitochondrial genome of Gadidae species, and showed a bootstrap value > 90% (based on 1000 replicates). Divergence time analysis showed that Gadidae species shared a common ancestor approximately 25.90 Mya (Figure 7). And the divergence time of seven lineages were mainly concentrated 0.07–11.95 Mya. The genus *Theragra* and *Gadus* displayed the closest genetic relationship and diverged 5.69 Mya. Furthermore, *G. macrocephalus* underwent genetic divergence, and *G. morhua* diverged 3.59 Mya. The sample of *G. macrocephalus* from China clustered with samples of *G. macrocephalus* from South Korea and Canada, and their divergence time was 0.07 Mya.

**Figure 8:**
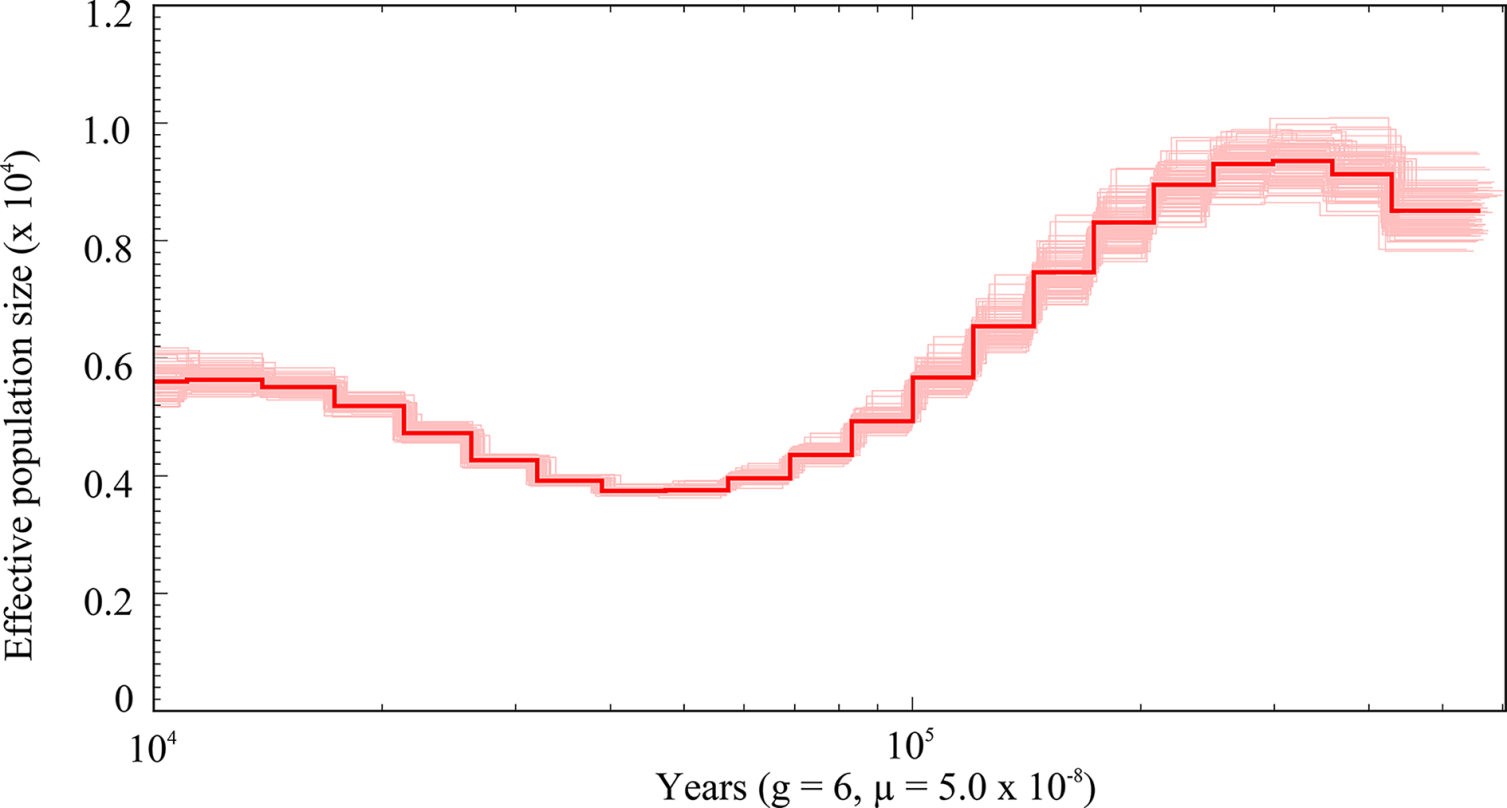
Demographic history analysis of *G. macrocephalus* Demographic history analysis of *G. macrocephalus* (thick line) and the 100 bootstrap replicates (thin lines) inferred from the whole-genome sequence data. The *x*-axis represents years (g: generation time, μ: mutation rate), and the *y*-axis indicates the effective population size of *G. macrocephalus*.

The PSMC model was used to infer historical changes in *N_e_* (Figure 8). The BWA results indicated that the rate of comparison was 96.76%, and the PSMC results showed a relatively stable *N_e_*. Demographic history analysis revealed that *G. macrocephalus* experienced a bottleneck effect from 300,000 to 40,000 years ago. Subsequently, the population experienced significant expansion between 40,000 and 15,000 years ago.

## Discussion

### Genome assembly and identification of microsatellite motifs

The order Gadiformes species include some of the most important commercial fish (e.g., *G. morhua*, *G. macrocephalus*) in the world and account for approximately 18% of the world’s total marine fish catch [27]. So far, the only available high-quality genome sequences of the order Gadiformes species are for *G. morhua* [28], *M. aeglefinus* [29], *G. chalcogrammus* (walleye pollock) [30] and *Lota lota* (burbot) [31]. Additionally, in the genus *Gadus*, the genomic data of *G. morhua* are relatively comprehensive [28,32]. However, very limited genomic researches have been focused on *G. macrocephalus* both at home and abroad.

In the present study, WGS was applied to preliminarily reveal the genomic data of *G. macrocephalus*. The genome size of *G. macrocephalus* was relatively small. Similarly, the genome sizes of the other fishes of the order Gadiformes species were less than 1 Gb: *G. morhua* (830 Mb) [28], *M. aeglefinus* (653 Mb) [29], *G. chalcogrammus* (683.61 Mb) [30] and *Lota lota* (576 Mb) [31]. The repetitive sequence content of *G. macrocephalus* was similar to the repetitive sequence content of other Gadidae species, such as *G. morhua* (25.40%) [28] and *G. chalcogrammus* (38.89%) [30]. But *Lota lota* (66.74%) [31] had higher repetitive sequence content. Repetitive sequences not only affect the structure of chromosomes but also control genomic evolution and recombination [33]. Further investigation is required to understand the functions of repetitive sequences in *G. macrocephalus*. For a genome assembly, if the heterozygosity is less than 1%, the genome is relatively effortless to assemble [17]. Therefore, for *G. macrocephalus*, the slightly low proportion of heterozygosity reflected that the genome assembly is relatively easy. Overall, combined with the heterozygosity and repetitive sequence content being slightly low, the small genome will make genome assembly easier in subsequent studies.

This is the first time that the genome sequences of *G. macrocephalus* were assembled. For the genome sequences of *G. macrocephalus*, the lengths of N50 contigs and scaffolds were short. A genome assembly would have high utility with an N50 contig size > 30 kb and an N50 scaffold size > 250 kb [34]. According to the criteria, the assembly quality was low in the present study. In the process of genome assembly, we selected *K*-mer (*K* = 19) possibly led to the low assembly quality [17]. Considering the low assembly quality, the gene prediction and annotation of genome sequences were not included, these genome sequences were only used to identify SSRs in the study. Studies have indicated that a higher-quality, chromosome-level genome assembly provides more accurate information for biological research [11,12]. The strategy of Illumina combined with third-generation and Hi-C techniques [35–37] should be applied to obtain a higher-quality genome of *G. macrocephalus*.

Recently, SSRs, which are chromosome-specific and frequently inherited in a Mendelian codominant fashion, have become the most commonly used molecular markers [38]. Many researchers have used DNA microsatellite loci to study the genetic diversity of *G. macrocephalus* [39,40]. In the present study, the frequency of microsatellites in the *G. macrocephalus* genome was very high (1497.5 microsatellites per Mb), which was markedly higher than that reported in *Sebastiscus albofasciatus* (556.8 microsatellites per Mb) [13] and *Tor tambra* (971.39 microsatellites per Mb) [14]. Among the motif types of microsatellites, the dinucleotide repeats were the most abundant because the number of repetitions is inversely proportional to the length of repetitions [41]. To ensure the usability of the microsatellite markers developed to date, subsequent validation studies are required.

### Mitochondrial genome assembly, phylogenetic and demographic history analysis

Complete mitochondrial genome is a powerful and popular tool to unravel the higher-level relationships of teleosts [42]. Hence, we constructed the complete mitochondrial genome of *G. macrocephalus* to further decipher its taxonomic status and systematic evolution. The genome organization and gene composition of the mitochondrial genome of *G. macrocephalus* were consistent with the results of a previous study using fish-versatile PCR primers [10]. The AT content was higher than the GC content in the mitochondrial genome of *G. macrocephalus*, similar to patterns reported in most vertebrates [43]. Besides, we further provided structural annotation of the mitochondrial genome of *G. macrocephalus.* Through the structural annotation of mitochondrial genome, we obtained the detailed information of genetic distribution and genomic structure, which could provide important raw materials for functional annotation and evolutionary analysis.

The phylogenetic tree showed that the phylogenetic relationships and divergence times among Gadidae species. 0.07–11.95 Mya, the diversity of seven lineages became increasingly abundant because climatic conditions were warmer and wetter [44]. Importantly, *G. macrocephalus* and *G. morhua* diverged 3.59 Mya, subsequently, *G. macrocephalus* from three different places diverged 0.07 Mya. Quaternary climate oscillations and geographical heterogeneity play important roles in determining species and genetic diversity distribution patterns [45], but how these factors affect the divergence of *G. macrocephalus* at the population level remains poorly understood. More efforts are still to be made in the further research on the divergence of this species.

Demographic history can be used to clarify the impact of geological and climate changes on the current species distribution, and help to formulate reasonable and effective strategies to preserve endangered species [46]. The present genetic structure of populations, species and communities has been mainly formed by Pleistocene climatic fluctuations [47]. Many marine fish species experienced bottlenecks and expansions in response to climate changes during the Pleistocene [48,49]. In the present study, reconstructing the Pleistocene demographic history of *G. macrocephalus*, revealed that *G. macrocephalus* population also experienced bottleneck and expansion. Similarly, Canino et al. reported that *G. macrocephalus* expansions were evident by using the mismatch distributions and Bayesian skyline plots of mitochondrial genome, but the start of expansions appeared to predate the last glacial maximum (21,000 years ago) [26]. This finding was consistent with our results. The results of demographic history analysis have important implications for the fishery management and conservation efforts of *G. macrocephalus*.

## Conclusions

In summary, the first whole-genome survey of *G. macrocephalus* was performed based on WGS data. The present study uncovered genomic characteristics of *G. macrocephalus*, including small genome, slightly low heterozygosity and repetitive sequence content. SSRs were identified from the genomic data, which could provide novel insight into population genetics and germplasm resource conservation in the future. Besides, the evolutionary relationships and divergence times of Gadidae species were determined, and the demographic history of *G. macrocephalus* was reconstructed. To improve the assembly quality of *G. macrocephalus* genome, we suggest that high-quality genome sequences should be generated by using third-generation sequencing and Hi-C techniques in the future.

## Data Availability

The sequence data were deposited in the Short Read Archive (SRA) databank (http://www.ncbi.nlm.nih.gov/sra) and are available under accession number PRJNA793360. The mitochondrial genome sequence was deposited in GenBank database with accession number OM174271.

## Abbreviations

BWA: Burrows–Wheeler Aligner
Mya: million years ago
*N _e_*: effective population size
PSMC: pairwise sequentially Markovian coalescent
SRA: Short Read Archive
SSR: simple sequence repeat
WGS: whole-genome sequencing

## References

[1] Hart J.L. (1973) Pacific fishes of Canada. Bull. Fish. Res. Board Can. 47, 180–730

[2] Grant W.S., Chang I.Z., Kobayashi T. and Stahl G. (1987) Lack of genetic stock discretion in Pacific cod (*Gadus macrocephalus*). Can. J. Fish. Aquat. Sci. 44, 490–498 10.1139/f87-061

[3] Li Z.L., Jin X.S., Zhang B., Zhou Z.P., Dan X.J. and Dai F.Q. (2012) Inter-annual variation of population characteristics of *Gadus macrocephalus* in the Yellow Sea. Oceanol. Limnol. Sin. 43, 924–931

[4] Wu D., Li J.C., Ye Z.J., Wang B., Liu S.D., Dong X.Q. et al. (2020) Growth and distribution characteristics of juvenile Pacific cod in the Yellow Sea. J. Ocean Univ. China 50, 63–73

[5] Hu B.Y., Chen B., Mao M.G., Chen M.K., Liu X., Cui Q.J. et al. (2018) Molecular characterization and expression analysis of the interleukin 1b gene in Pacific cod (*Gadus macrocephalus*). Dev. Comp. Immunol. 88, 213–218 10.1016/j.dci.2018.07.025 30048700

[6] Bryan D.R., McDermott S.F., Nielsen J.K., Fraser D. and Rand K.M. (2021) Seasonal migratory patterns of Pacific cod (*Gadus macrocephalus*) in the Aleutian Islands. Anim. Biotelem. 9, 24 10.1186/s40317-021-00250-2

[7] Smirnova M.A., Orlova S.Y. and Orlov A.M. (2019) The population genetic organization of pacific cod *Gadus macrocephalus* in the North Pacific based on microsatellite analysis. J. Ichthyol. 59, 555–565 10.1134/S0032945219040155

[8] Choi D.H., Sohn M.H., Kim M.J. and Lee S.J. (2019) Feeding habits of Pacific cod (*Gadus macrocephalus*) in the west coast of Yellow Sea of Korea. Korean J. Ichthyol. 31, 77–82 10.35399/ISK.31.2.2

[9] Jiang J.L., Xu J., Liu Y.M., Mao Y.X., Wang H.S., Sun Y. et al. (2022) Immune response of pacific cod larvae stimulated with PolyI:C. Aquac. Res. 53, 3392–3400 10.1111/are.15847

[10] Song H.Y., Hyun Y.S., Woo J., Oh S. and An H.S. (2016) Complete mitochondrial genome of the Pacific cod, *Gadus macrocephalus* (Gadiformes, Gadidae). Mitochondrial DNA B Resour 1, 829–830 10.1080/23802359.2016.1247668 33473643 PMC7800020

[11] Meyer A., Schloissnig S., Franchini P., Du K., Woltering J.M., Irisarri I. et al. (2021) Giant lungfish genome elucidates the conquest of land by vertebrates. Nature 590, 284–289 10.1038/s41586-021-03198-8 33461212 PMC7875771

[12] Thompson A.W., Hawkins M.B., Parey E., Wcisel D.J., Ota T. and Kawasaki K. (2021) The bowfin genome illuminates the developmental evolution of ray-finned fishes. Nat. Genet. 53, 1373–1384 10.1038/s41588-021-00914-y 34462605 PMC8423624

[13] Jia C.H., Yang T.Y., Yanagimoto T. and Gao T.X. (2021) Comprehensive draft genome analyses of three rockfishes (Scorpaeniformes, *Sebastiscus*) via Genome Survey Sequencing. Curr. Issues Mol. Biol. 43, 2048–2058 10.3390/cimb43030141 34889891 PMC8929126

[14] Surachat K., Deachamag P. and Wonglapsuwan M. (2022) The first de novo genome assembly and sex marker identification of Pluang Chomphu fish (*Tor tambra*) from Southern Thailand. Comput. Struct. Biotechnol. J. 20, 1470–1480 10.1016/j.csbj.2022.03.021 35422970 PMC8976102

[15] Li H. and Durbin R. (2011) Inference of human population history from individual whole-genome sequences. Nature 475, 493–496 10.1038/nature10231 21753753 PMC3154645

[16] Chen S.F., Zhou Y.Q., Chen Y. and Gu J. (2018) Fastp: an ultra-fast all-in-one FASTQ preprocessor. Bioinformatics 34, i884–i890 10.1093/bioinformatics/bty560 30423086 PMC6129281

[17] Marcais G. and Kingsford C. (2011) A fast, lock-free approach for efficient parallel counting of occurrences of k-mers. Bioinformatics 27, 764–770 10.1093/bioinformatics/btr011 21217122 PMC3051319

[18] Luo R.B., Liu B.H., Xie Y.L., Li Z.Y., Huang W.H., Yuan J.Y. et al. (2012) SOAPdenovo2: an empirically improved memory-efficient short-read de novo assembler. Gigascience 1, 18 10.1186/2047-217X-1-18 23587118 PMC3626529

[19] Beier S., Thiel T., Münch T., Scholz U. and Mascher M. (2017) MISA-web: a web server for microsatellite prediction. Bioinformatics 33, 2583–2585 10.1093/bioinformatics/btx198 28398459 PMC5870701

[20] Allio R., Schomaker-Bastos A., Romiguier J., Prosdocimi F., Nabholz B. and Delsuc F. (2020) MitoFinder: efficient automated large-scale extraction of mitogenomic data in target enrichment phylogenomics. Mol. Ecol. Resour. 20, 892–905 10.1111/1755-0998.13160 32243090 PMC7497042

[21] Li D., Liu C.M., Luo R., Sadakane K. and Lam T.W. (2015) MEGAHIT: an ultra-fast single-node solution for large and complex metagenomics assembly via succinct de Bruijn graph. Bioinformatics 31, 1674–1676 10.1093/bioinformatics/btv033 25609793

[22] Iwasaki W., Fukunaga T., Isagozawa R., Yamada K., Maeda Y., Satoh T.P. et al. (2013) MitoFish and MitoAnnotator: a mitochondrial genome database of fish with an accurate and automatic annotation pipeline. Mol. Biol. Evol. 30, 2531–2540 10.1093/molbev/mst141 23955518 PMC3808866

[23] Tamura K., Stecher G. and Kumar S. (2021) MEGA11: Molecular Evolutionary Genetics Analysis Version 11. Mol. Biol. Evol. 38, 3022–3027 10.1093/molbev/msab120 33892491 PMC8233496

[24] Kumar S., Stecher G., Suleski M. and Hedges S.B. (2017) TimeTree: a resource for timelines, timetrees, and divergence times. Mol. Biol. Evol. 34, 1812–1819 10.1093/molbev/msx116 28387841

[25] Li H. and Durbin R. (2009) Fast and accurate short read alignment with Burrows-Wheeler Transform. Bioinformatics 25, 1754–1760 10.1093/bioinformatics/btp324 19451168 PMC2705234

[26] Canino M.F., Spies I.B., Cunningham K.M., Hauser L. and Grant W.S. (2010) Multiple ice-age refugia in Pacific cod, *Gadus Macrocephalus*. Mol. Ecol. 19, 4339–4351 10.1111/j.1365-294X.2010.04815.x 20819160

[27] FAO. (2004) The State of world fisheries and aquaculture (SOFIA) 2004. https://www.fao.org/3/y5600e/y5600e00.htm

[28] Star B., Nederbragt A.J., Jentoft S., Grimholt U., Malmstrøm M., Gregers T.F. et al. (2011) The genome sequence of Atlantic cod reveals a unique immune system. Nature 477, 207–210 10.1038/nature10342 21832995 PMC3537168

[29] Tørresen O.K., Brieuc M.S.O., Solbakken M.H., Sørhus E., Nederbragt A.J., Jakobsen K.S. et al. (2018) Genomic architecture of haddock (*Melanogrammus aeglefinus*) shows expansions of innate immune genes and short tandem repeats. BMC Genomics 19, 240 10.1186/s12864-018-4616-y 29636006 PMC5894186

[30] Noh E.S., Kang B.C., Kim J., Jeon J.H., Kim Y.O., Byun S.G. et al. (2022) Draft assembled genome of Walleye Pollock (*Gadus chalcogrammus*). Front. Mar. Sci. 9, 744941 10.3389/fmars.2022.744941

[31] Han Z.Q., Liu M.H., Liu Q., Zhai H., Xiao S.J. and Gao T.X. (2021) Chromosome-level genome assembly of burbot (*Lota lota*) provides insights into the evolutionary adaptations in freshwater. Mol. Ecol. Resour. 00, 1–12 10.1111/1755-0998.13382 33730415

[32] Matschiner M., Barth J.M.I., Tørresen O.K., Star B., Baalsrud H.T., Brieuc M.S.O. et al. (2022) Supergene origin and maintenance in Atlantic cod. Nat. Ecol. Evol. 6, 469–481 10.1038/s41559-022-01661-x 35177802 PMC8986531

[33] Shapiro J.A. and von Sternberg R. (2005) Why repetitive DNA is essential to genome function. Biol. Rev. 80, 227–250 10.1017/S1464793104006657 15921050

[34] Hamilton J.P. and Buell C.R. (2012) Advances in plant genome sequencing. Plant J. 70, 177–190 10.1111/j.1365-313X.2012.04894.x 22449051

[35] Jain M., Koren S., Miga K.H., Quick J., Rand A.C., Sasani T.A. et al. (2018) Nanopore sequencing and assembly of a human genome with ultra-long reads. Nat. Biotechnol. 36, 338–345 10.1038/nbt.4060 29431738 PMC5889714

[36] Wenger A.M., Peluso P., Rowell W.J., Chang P.C., Hall R.J., Concepcion G.T. et al. (2019) Accurate circular consensus long-read sequencing improves variant detection and assembly of a human genome. Nat. Biotechnol. 37, 1155–1162 10.1038/s41587-019-0217-9 31406327 PMC6776680

[37] Lieberman-Aiden E., van Berkum N.L., Williams L., Imakaev M., Ragoczy T., Telling A. et al. (2009) Comprehensive mapping of long-range interactions reveals folding principles of the human genome. Science 326, 289–293 10.1126/science.1181369 19815776 PMC2858594

[38] Desai H., Hamid R., Ghorbanzadeh Z., Bhut N., Padhiyar S.M., Kheni J. et al. (2021) Genic microsatellite marker characterization and development in little millet (*Panicum sumatrense*) using transcriptome sequencing. Sci. Rep. 11, 20620 10.1038/s41598-021-00100-4 34663808 PMC8523711

[39] Song N., Liu M., Yanagimoto T., Sakurai Y., Han Z.Q. and Gao T.X. (2016) Restricted gene flow for *Gadus macrocephalus* from Yellow Sea based on microsatellite markers: geographic block of Tsushima current. Int. J. Mol. Sci. 17, 467–477 10.3390/ijms17040467 27043534 PMC4848923

[40] Stroganov A.N. (2013) Formation of genetic diversity in populations of Pacific cod (*Gadus macrocephalus* Tilesius) (Gadidae). Genetika 49, 1300–1305 10.1134/S102279541309010X 25470931

[41] Chen M., Tan Z., Zeng G. and Peng J. (2010) Comprehensive analysis of simple sequence repeats in pre-miRNAs. Mol. Biol. Evol. 27, 2227–2232 10.1093/molbev/msq100 20395311

[42] Miya M., Kawaguchi A. and Nishida M. (2001) Mitogenomic exploration of higher teleostean phylogenies: a case study for moderate-scale evolutionary genomics with 38 newly determined complete mitochondrial DNA sequences. Mol. Biol. Evol. 18, 1993–2009 10.1093/oxfordjournals.molbev.a003741 11606696

[43] Saccone C., De Giorgi C., Gissi C., Pesole G. and Reyes A. (1999) Evolutionary genomics in Metazoa: the mitochondrial DNA as a model system. Gene 238, 195–209 10.1016/S0378-1119(99)00270-X 10570997

[44] Aduse-Poku K., van Bergen E., Sáfián S., Collins S.C., Etienne R.S., Herrera-Alsina L. et al. (2021) Miocene climate and habitat change drove diversification in bicyclus, Africa’s largest radiation of satyrine butterflies. Syst. Biol. 71, 570–588 10.1093/sysbio/syab066 PMC9016770 34363477

[45] Xue C., Geng F.D., Li J.J., Zhang D.Q., Gao F., Huang L. et al. (2021) Divergence in the Aquilegia ecalcarata complex is correlated with geography and climate oscillations: evidence from plastid genome data. Mol. Ecol. 30, 5796–5813 10.1111/mec.16151 34448283

[46] Karamanlidis A.A., Skrbinšek T., Amato G., Dendrinos P., Gaughran S., Kasapidis P. et al. (2021) Genetic and demographic history define a conservation strategy for earth's most endangered pinniped, the Mediterranean monk seal Monachus monachus. Sci. Rep. 11, 373 10.1038/s41598-020-79712-1 33431977 PMC7801404

[47] Hewitt G. (2000) The genetic legacy of the quaternary ice ages. Nature 405, 907–913 10.1038/35016000 10879524

[48] Carr S.M. and Marshall H.D. (2008) Intraspecific phylogeographic genomics from multiple complete mtDNA genomes in Atlantic cod (*Gadus morhua*): origins of the “codmother,” transatlantic vicariance and midglacial population expansion. Genetics 180, 381–389 10.1534/genetics.108.089730 18716332 PMC2535689

[49] Delrieu-Trottin E., Hubert N., Giles E.C., Chifflet-Belle P., Suwalski A. et al. (2020) Coping with pleistocene climatic fluctuations: demographic responses in remote endemic reef fishes. Mol. Ecol. 29, 2218–2233 10.1111/mec.15478 32428327

